# Social engagement and allostatic load mediate between adverse childhood experiences and multimorbidity in mid to late adulthood: the Canadian Longitudinal Study on Aging

**DOI:** 10.1017/S0033291721003019

**Published:** 2023-03

**Authors:** Leslie Atkinson, Divya Joshi, Parminder Raina, Lauren E. Griffith, Harriet MacMillan, Andrea Gonzalez

**Affiliations:** 1 Department of Psychology, Ryerson University, Toronto, Canada; 2 Department of Health Research Methods, Evidence, and Impact, McMaster University, Hamilton, Canada; 3 Department of Psychiatry & Behavioural Neurosciences, McMaster University, Hamilton, Canada

**Keywords:** Adverse childhood experiences, social engagement, allostatic load, multimorbidity

## Abstract

**Background:**

Adverse childhood experiences (ACEs) are associated with multimorbidity in adulthood. This link may be mediated by psychosocial and biological factors, but evidence is lacking. The current study evaluates this mediation model.

**Method:**

We analyzed data from the Canadian Longitudinal Study of Aging (*N* = 27 170 community participants). Participants were 45–85 years at recruitment, when allostatic load and social engagement data were collected, and 3 years older at follow-up, when ACEs and multimorbidity data were collected. Structural equation modeling was used to test for mediation in the overall sample, and in sex- and age-stratified subsamples, all analyses adjusted for concurrent lifestyle confounds.

**Results:**

In the overall sample, ACEs were associated with multimorbidity, directly, *β* = 0.12 (95% confidence interval 0.11–0.13) and indirectly. Regarding indirect associations, ACEs were related to social engagement, *β* = −0.14 (−0.16 to −0.12) and social engagement was related to multimorbidity, *β* = −0.10 (−0.12 to −0.08). ACEs were related to allostatic load, *β* = 0.04 (0.03–0.05) and allostatic load was related to multimorbidity, *β* = 0.16 (0.15–0.17). The model was significant for males and females and across age cohorts, with qualifications in the oldest stratum (age 75–85).

**Conclusions:**

ACEs are related to multimorbidity, directly and via social engagement and allostatic load. This is the first study to show mediated pathways between early adversity and multimorbidity in adulthood. It provides a platform for understanding multimorbidity as a lifespan dynamic informing the co-occurrence of the varied disease processes represented in multimorbidity.

Multimorbidity involves the co-occurrence of at least two chronic health conditions, with comorbidities unrelated to an index diagnosis (Nguyen et al., 2019; Wang, Si, Cocker, Palmer, & Sanderson, 2018; Yurkovich, Avina-Zubieta, Thomas, Gorenchtein, & Lacaille, 2015). It is the latter clause that impedes understanding of this condition. What common dynamics underlie the seemingly distinct disease processes involved? This nescience compounds intervention complexity, foments piecemeal service provision, exacerbates treatment burden, and undermines patient compliance (Gallacher et al., 2014). Rates of multimorbidity are high worldwide, particularly in the 65+ age group (Nguyen et al., 2019), although absolute numbers are greater amongst those under age 65, due to population age distribution (Sheikh et al., 2016). Furthermore, rates are escalating (Steffler et al., 2021; Uijen & van de Lisdonk, 2008; Ward & Schiller, 2013), and climate change may further complicate multimorbidity patterns (Thienemann, Ntusi, Battegay, Mueller, & Cheetham, 2020). Multimorbidity is burdensome (Sheikh et al., 2016) and costly (Wang et al., 2018). Management of multimorbidity itself carries risks (Sheikh et al., 2016), and the condition is strongly related to age at mortality (Dugravot et al., 2020). Not surprisingly, multimorbidity is considered a major challenge of the 21st century [Institute of Medicine, 2012; World Health Organization (WHO), 2008].

There is evidence that multimorbidity is influenced by adverse childhood experiences (ACEs; Felitti et al., 1998; Tomasdottir et al., 2015), defined as potentially traumatic childhood events or environmental aspects that undermine the child's sense of safety (Centers for Disease Control and Prevention, 2019). ACEs, individually and in combination, are linked to the delay of early developmental milestones (Jensen, Berens, & Nelson, 2017), early- and late-life psychiatric outcomes (Gershon, Sudheimer, Tirouvanziam, Williams, & O'Hara, 2013; Taylor & Rogers, 2005), chronic physical disease, and premature mortality (Berens, Jensen, & Nelson, 2017). Moreover, the more numerous the ACEs, the greater the number of adverse outcomes experienced by the individual (Anda et al., 2006; Atkinson et al., 2015). Thus, the number of adversities is linked to the accumulation of simultaneous cognitive, mood, lifestyle (smoking, school dropout, and arrest), and physical disease outcomes in early adulthood (Atkinson et al., 2015), and to the comorbidity of outcomes, including physical health, mental health, substance abuse, memory, and interpersonal difficulties in mid to late adulthood (Anda et al., 2006). The power and latitude of empirical findings suggest that cumulated ACEs may offer deep insights into coordinated developmental trajectories that encompass diverse outcomes (Atkinson et al., 2015). In particular, they may help explain the recalcitrant complexities of multimorbidity.

Investigators propose two mediation models to explain the long-term consequences of early adversity. One involves chains of psychosocial risk: early environmental adversity activates a chain reaction in which one negative factor activates another (Rutter, 1989; Turner, Thomas, & Brown, 2016). Other investigators propose that biological embedding mediates the link between early adversity and subsequent outcomes (Anda et al., 2006; Belsky et al., 2017; Danese & McEwen, 2012; Evans, Li, & Whipple, 2013). ACEs activate physiological stress in a roughly dose-response manner, thereby undermining the development of diverse biological functions. This embedded risk, with its pervasive influence, magnifies the probability of multiple, seemingly unrelated comorbidities across psychosocial, functional, and physical health domains, and carries this risk across the lifespan. Neither model has been empirically validated (Atkinson et al., 2015; Evans et al., 2013; Nelson et al., 2020; Turner et al., 2016).

Although ‘sharply contrasting’ (Turner et al., 2016), the psychosocial chain and biological embedding models are not mutually exclusive; both dynamics may link ACEs to cumulative outcome (Atkinson et al., 2015). Presumably, many features serve to mediate the relation between ACEs and outcomes, but Atkinson et al. (2015) emphasize the criterion of parsimony. They proposed that putative mediators be associated with diverse risk factors and with diverse outcome factors, and with the sum of each. Further, the mediators must be programmed relatively early in life and show some stability across time.

By way of potential environmental mediators, Atkinson et al. (2015) posited psychosocial variables such as attachment security as an early form of social engagement. Attachment is related to numerous outcomes spanning diverse psychological and physical health domains (Carter, 2017; Maunder & Hunter, 2015) and meets early programming and stability criteria (Fraley, 2002). Attachment security is also related to the accumulation of early adversities (Belsky & Fearon, 2002). Of import here, attachment was posited as an exemplar of the broader construct of relationality because life course models of human development universally incorporate the relational dynamic (Atkinson, 2019). This is so for psychosocial (Ainsworth, Blehar, Waters, & Wall, 1978), sociocultural/cognitive (Fogel, 1993; Vygotsky, 1978), neurophysiological (Gunnar, Hostinar, Sanchez, Tottenham, & Sullivan, 2015; McEwen & Wingfield, 2003), psycho-evolutionary (Slavin & Kriegman, 1992), and biological-evolutionary (Trivers, 1974) theories. The relational imperative conforms to the biology of an altricial species wherein the young are utterly dependent on adult caregivers (Atkinson, 2019). The impact of relationality is profound: It shapes and constrains the interpretation of context. Indeed, the meaning is ‘not entirely ‘in’ the self or ‘in’ the other’; it is intersubjective and ‘becomes available’ via active engagement (Fogel, 1993) as ‘the property of a dyad, not an individual’ (Hilburn-Cobb, 2004). Significant in the current context, ‘health happens between people’ (Maunder & Hunter, 2015). Thus, more engaging maternal communication regarding diet is linked to better dietary adherence and medical outcomes (Chisholm et al., 2014a), and fewer behavioral difficulties (Chisholm, Gonzalez, & Atkinson, 2014b) in young children with type I diabetes. Similarly, insecure attachment is associated with poorer type 2 diabetes management and more negative disease outcomes (Ciechanowski et al., 2004) in adulthood. Adult attachment is also associated with poorer self-management among patients with multimorbidity (Brenk-Franz et al., 2015), and this association is mediated by the patient–provider relationship (Brenk-Franz et al., 2017). The centrality of social engagement to human development, healthy and otherwise, suggests that it is likely an important mediator between ACEs and multimorbidity.

In terms of a biological mechanism, and in the context of multiple, simultaneous risks and coinciding adverse outcomes, the mediating role of allostatic load has been hypothesized (Anda et al., 2006; Atkinson et al., 2015; Belsky et al., 2017). Allostasis involves the active adjustment of physiological function in adaptive anticipation of environmental change. There is a cumulative physiological cost to allostasis, however, termed ‘allostatic load’ (Schulkin & Sterling, 2019). If environmental challenges are repeated and/or chronic, cumulative physiological dysregulation, or ‘allostatic overload’ develops across multiple systems that support allostasis (McLoughlin, Kenny, & McCrory, 2020). Allostatic overload undermines physiological flexibility, thereby potentiating varied maladaptive outcomes across cognitive, psychiatric, and physical domains (Evans, 2003; McEwen, 2000). Consistent with the criteria for the parsimonious selection of mediators (Atkinson et al., 2015), physiological stress responses are programmed early in life with enduring effects (O'Connor, 2015; Teicher et al., 2003). Moreover, allostatic load increases with levels of cumulative adversity exposure. Thus, Evans and coworkers (Belsky et al., 2017; Evans, 2003; Evans, Kim, Ting, Tesher, & Shannis, 2007) showed that cumulative physical and psychosocial risk exposure in 8- to 10-year-old children was positively related to cumulative allostatic load [consisting of summed cortisol, epinephrine and norepinephrine, resting diastolic and systolic blood pressure, and body mass index (BMI)] assessed 3–4 years later. Belsky et al. (2017) showed that the cumulation of childhood risk factors prospectively predicts cumulative allostatic load (as assessed with an index consisting of 18 biomarkers related to cardiovascular, metabolic, endocrine, pulmonary, hepatic, renal, immune, and periodontal systems) at ages 26–38. Furthermore, Belsky et al. (2017) replicated these findings with participants' retrospective personal history reports in adulthood. A recent review (Guidi, Lucente, Sonino, & Fava, 2021) showed that allostatic load is linked to numerous chronic disorders, including cardiovascular disease, cancer, diabetes, periodontal disease, mood and anxiety disorders, post-traumatic stress disorders, psychoses, and alcohol dependence.

There are theoretical reasons to believe that ACEs are linked to multimorbidity via social engagement and allostatic load, and such data as exist are consistent with this model. However, to our knowledge, these relations have not been empirically validated in a coherent model. We aim to validate the full model here.

## Methodology

### Study design and population

**Fig. 1.**
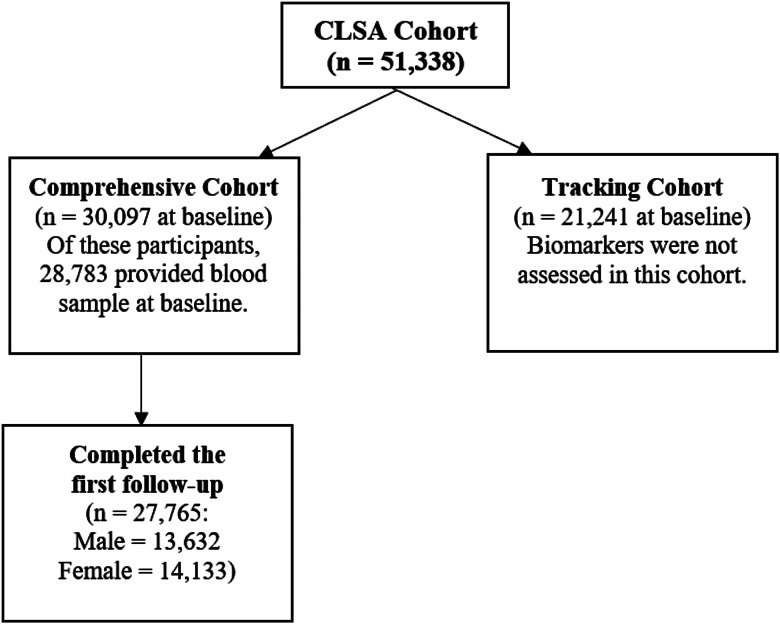
Process of selection of study participants.

The Canadian Longitudinal Study on Aging (CLSA) is a national, population-based, longitudinal study involving a stratified random sample of 51 338 community-dwelling participants aged 45–85 years at recruitment (Raina et al., 2009, 2019). Residents in the Canadian territories, on First Nation reserves, or in nursing homes, full-time members of the armed forces, or individuals with significant cognitive impairment were excluded from study participation. Physiological measures were collected on a subsample, the Comprehensive Cohort, which included 30 097 participants at recruitment, randomly selected within sex and age strata among individuals residing within 25–50 km of a CLSA data collection site in 11 locations across Canada. Of these participants, 27 170 (90.3%) and 28 783 (95.6%) provided blood and urine samples, respectively (see Fig. 1 for flow chart of study participant selection process). This sample included 14 133 (52.3%) females and 13 632 (49.7%) males. Information was collected through in-home interviews and participants visited research sites for collection of physical and biological measures. For the present study, we analyzed baseline data (collected September 2011 to May 2015) pertaining to allostatic load and social engagement, and first follow-up data (July 2015 to July 2018) pertaining to ACEs and multimorbidity.

### Study measures

#### Adverse childhood experiences (ACEs)

A questionnaire consisting of 14 items adapted from the Childhood Experiences of Violence Questionnaire (CEVQ) (Tanaka et al., 2012; Walsh, MacMillan, Trocmé, Jamieson, & Boyle, 2008) and the National Longitudinal Study of Adolescent to Adult Health Wave III questionnaire (Harris & Udry, 2018) were used to measure exposure to ACEs before the age of 16. Frequency and severity of childhood exposure to physical, emotional, and sexual abuse, neglect, and intimate partner violence were assessed on an ordinal scale (never, 1–2 times, 3–5 times, 6–10 times, or more than 10 times) and responses were dichotomized in accordance with the CEVQ guidelines to identify the presence or absence of the exposure. Physical abuse was considered present if the participant endorsed being: (1) slapped on the face, head or ears, or hit or spanked with something hard three or more times; (2) pushed, grabbed, or shoved, or having something thrown to hurt three or more times; or (3) kicked, bit, punched, choked, burned, or physically attacked in some other way one or more times (Tanaka et al., 2012). Sexual abuse was considered present if the participant reported being sexually touched against their will or being threatened or forced into unwanted sexual activity one or more times (Tanaka et al., 2012). Emotional abuse was present if the participant reported parents or guardians swearing, saying hurtful or insulting things that made them feel unloved or unwanted three or more times. Neglect was present if the participant reported parents or guardians not having taken care of their basic needs one or more times. Exposure to intimate partner violence was present if the participant saw or heard parents or guardians say hurtful things to each other six or more times or hit each other three or more times (Tanaka et al., 2012). Other forms of household adversity, such as parental divorce or separation, parental death, and living with a family member with mental health problems, were assessed dichotomously as ‘yes’ or ‘no’. An ACEs index was created by summing the individual ACEs that participants experienced (Cronbach's *α* = 0.73).

#### Allostatic load index

An allostatic load index was constructed to assess cumulative physiological dysregulation. Hematological, cardiometabolic, and biomarkers comprising white blood cells, HbA1c, albumin, alanine aminotransferase, creatinine, hemoglobin, ferritin, C-reactive protein, total cholesterol, high-density lipoprotein cholesterol, low-density lipoprotein cholesterol, triglycerides, systolic and diastolic blood pressure, heart rate, BMI, waist circumference, and waist to hip ratio comprised the allostatic load index. High risk was defined using the upper or lower 25th percentile of the sample distribution of the specific biomarker, consistent with recommendations (Seeman, Singer, Rowe, Horwitz, & McEwen, 1997). An allostatic load index was calculated by summing the number of biomarkers falling within the high-risk categories. This count-based formula is used most often in the literature (Gallo et al., 2012; Juster, McEwen, & Lupien, 2010) and is recommended to harmonize international work (McLoughlin et al., 2020).

#### Social engagement

Social engagement was assessed by combining social support and social participation measures. Social support was assessed with the 19-item Medical Outcomes Study and Social Support Survey, measuring perception of emotional support, instrumental assistance, information, guidance and feedback, personal appraisal support, and companionship (Sherbourne & Stewart, 1991). Scores were calculated by averaging responses and transforming them to range from 0 to 100 (Sherbourne & Stewart, 1991). Social participation was assessed as the frequency of participation (at least daily, weekly, monthly, or yearly) in community-related activity. Again, social support and social participation were combined into a single social engagement measure.

#### Multimorbidity

Based on the stability of prevalence estimates, Fortin, Stewart, Poitras, Almirall, and Maddocks (2012) recommended the inclusion of at least 12 chronic diseases in the construction of a multimorbidity index, each with high prevalence and impact or burden in the population. We constructed an index of 21 conditions meeting these conditions in North America. These involve skeletal, nervous, endocrine, cardiovascular, lymphatic, and respiratory organ systems, viz. heart disease, myocardial infarction, angina, stroke, transient ischemic attack, peripheral vascular disease, hypertension, diabetes, chronic obstructive pulmonary disease, Parkinson's disease, epilepsy, multiple sclerosis, migraines, osteoarthritis, osteoporosis, kidney disease, cataracts, glaucoma, cancer, mood disorders, and anxiety disorders. Participants reported chronic conditions diagnosed by a health professional with past and/or expected minimum duration of 6 months. The number of conditions endorsed was summed for each participant.

#### Covariates

To limit the possibility that relations between ACEs, mediators, and outcomes were confounded by adult life choices and circumstances (Turner et al., 2016), several covariates were incorporated. Cigarette smoking was assessed as never, occasional, or current smoker with 0–15 cigarettes/day, and current smoker with >15 cigarettes/day. Nutritional intake quality was assessed using the Seniors in the Community Questionnaire (Keller, Goy, & Kane, 2005). Meeting recommendations for fruit/vegetable intake is often used as an indicator of diet quality. Less than four daily servings of fruits/vegetables was considered unhealthy. Physical activity was assessed using the Physical Activity Scale for the Elderly scale (Washburn, Smith, Jette, & Janney, 1993). Participants were considered physically active if they engaged in moderate or vigorous activity three or more times/week. Participants also reported the frequency of alcohol consumption during the last 12 months on a scale of 0 (‘never’) to 7 (‘almost every day’). Annual household income was categorized as less than $20 000, $20 000–49 999, $50 000–99 999, $100 000–149 999, and $150 000 and above. Data on all covariates were collected at baseline visit. As a final check on the model, to ensure that results could not be solely attributed to the effect of baseline multimorbidity and allostatic load, rather than *vice versa*, baseline multimorbidity was also entered into the model as a covariate.

### Statistical analysis

Descriptive analysis was adjusted for the sampling design and performed using inflation weights provided by the CLSA (CLSA Methodology Working Group, 2017). Structural equation modeling with Full Information Maximum Likelihood (FIML) was used to test for mediation and manage missing data. Under the missing at random assumption, FIML estimates a likelihood function for each individual based on the observed variables, such that all available data are used in deriving unbiased parameter estimates and standard errors where data are missing (Enders & Bandalos, 2001; Wothke, 2000).

A latent variable for social engagement was created from social support and participation scales. The path models tested the direct effect of ACEs on allostatic load, social engagement, and multimorbidity, the direct effect of allostatic load and social engagement on multimorbidity, and the indirect effect of ACEs on multimorbidity via allostatic load index and social engagement. We included a covariance between the two mediators – social engagement and allostatic load – and tested the path from social engagement to allostatic load. Overall model and age- and sex-stratified models were tested. Age- and sex-stratified models were adjusted for annual income, physical activity, nutritional intake, and smoking, and the overall model was further adjusted for age and sex. Model fit was assessed using the Comparative Fit Index (CFI) value of >0.95, Standardized Root Mean Square Residual (SRMR) of >0.05, and the Root Mean Square Error of Approximation (RMSEA) of <0.05 indicate good fit. Analyses were conducted in SAS v.9.4.

## Results

**Fig. 2.**
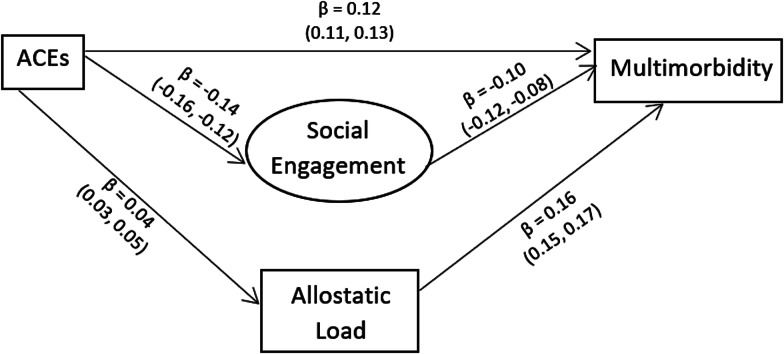
Structural model of factors influencing multimorbidity. Model adjusted for age, sex, income, smoking, nutrition, and alcohol consumption. Covariance between social engagement and allostatic load was included. All paths are statistically significant, p < .0001. ACEs = Adverse childhood experiences

Table 1 presents descriptive sample features. Figure 2 shows a path diagram of the structural model of factors associated with multimorbidity in the overall CLSA sample. Observed variables are denoted by rectangles, the latent construct by an ellipse. The RMSEA for the overall model was 0.06, SRMR was 0.02, and CFI was 0.97, indicating good fit of data with the hypothesized structural model. Fit indices for sex- and age-stratified models also indicate good data fit. In the overall sample, the *R*^2^ for multimorbidity indicates that ACEs, allostatic load, and social function together account for 26.5% of the variation in multimorbidity.

**Table 1.** Descriptive statistics by age and sex: adverse childhood experiences, multimorbidity, mediators, and covariates

	Overall sample	Ages 45–54		Ages 55–64		Ages 65–74		Ages 75–85	
		Male	Female	Male	Female	Male	Female	Male	Female
*N*	27 765	2141 (52.5)	2252 (47.5)	4379 (50.5)	4757 (49.5)	4114 (47.7)	4122 (52.3)	2998 (45.7)	3002 (54.3)
Total ACEs score, mean (s.e.)	1.4 (0.01)	1.6 (0.04)	1.7 (0.04)	1.4 (0.03)	1.7 (0.03)	1.2 (0.03)	1.4 (0.03)	1.0 (0.03)	1.0 (0.03)
ACEs index									
0	9845 (35.9)	688 (32.5)	673 (31.5)	1491 (35.0)	1462 (32.1)	1506 (38.0)	1454 (37.3)	1261 (44.8)	1310 (48.4)
1	7250 (26.9)	545 (26.1)	548 (24.7)	1185 (27.9)	1126 (24.5)	1152 (29.7)	1018 (26.0)	854 (30.8)	822 (29.1)
2	3950 (15.5)	331 (16.2)	326 (16.0)	669 (16.5)	745 (16.1)	580 (15.1)	579 (15.0)	389 (13.9)	331 (11.5)
3 +	5226 (21.8)	493 (25.2)	601 (27.8)	847 (20.6)	1202 (27.3)	644 (17.2)	814 (21.7)	290 (10.5)	335 (11.0)
Multimobidity, mean (s.e.)	2.4 (0.02)	1.2 (0.04)	1.6 (0.04)	1.8 (0.03)	2.3 (0.04)	2.8 (0.04)	3.4 (0.05)	3.8 (0.05)	4.5 (0.06)
Multimorbidity, *n* (%)									
0	3733 (19.3)	744 (36.9)	582 (27.9)	967 (25.6)	722 (18.5)	373 (10.8)	218 (5.9)	88 (3.4)	39 (1.8)
1	4952 (22.8)	591 (30.7)	576 (28.4)	1084 (27.9)	1013 (24.0)	721 (19.9)	525 (15.3)	269 (10.6)	173 (7.0)
2	4686 (19.2)	355 (18.0)	422 (21.1)	797 (20.0)	895 (20.6)	765 (20.6)	697 (18.8)	459 (18.3)	296 (11.8)
3+	11 157 (38.7)	289 (14.5)	480 (22.6)	1130 (26.4)	1682 (36.9)	1768 (48.7)	2205 (60.0)	1693 (67.8)	1910 (79.3)
Allostatic load index, mean (s.e.)	4.2 (0.02)	5.2 (0.08)	2.4 (0.06)	5.5 (0.06)	3.0 (0.05)	5.3 (0.06)	3.5 (0.05)	4.8 (0.06)	3.4 (0.05)
Social participation, mean (s.e.)	3.0 (0.01)	2.9 (0.02)	3.0 (0.01)	2.9 (0.01)	3.0 (0.01)	3.0 (0.01)	3.1 (0.01)	3.0 (0.01)	3.1 (0.01)
Social support, mean (s.e.)	82.6 (0.10)	83.0 (0.40)	84.9 (0.33)	82.5 (0.30)	83.1 (0.27)	83.1 (0.30)	81.8 (0.29)	81.4 (0.36)	77.7 (0.37)
Total annual household income, *n* (%)									
<$ 20 000	1303 (4.2)	55 (2.6)	80 (3.0)	150 (3.2)	211 (4.1)	148 (3.2)	284 (6.8)	92 (3.1)	283 (11.2)
$20 000–<50 000	5617 (17.8)	166 (8.3)	228 (9.3)	475 (10.3)	735 (14.5)	804 (20.2)	1257 (32.1)	765 (29.0)	1187 (44.3)
$50 000–<100 000	9286 (33.5)	529 (25.3)	665 (29.9)	1242 (28.4)	1536 (34.2)	1666 (42.5)	1511 (40.9)	1276 (44.0)	861 (32.6)
$100 000–<150 000	5218 (22.6)	593 (29.5)	569 (27.7)	1132 (27.5)	1033 (24.1)	772 (19.7)	475 (13.3)	439 (15.5)	205 (8.7)
$150 000 or more	4617 (21.8)	731 (34.2)	617 (30.1)	1219 (30.6)	975 (23.1)	520 (14.4)	254 (6.9)	227 (8.4)	74 (3.2)
Low physical activity, *n* (%)	20 585 (72.9)	1415 (66.5)	1586 (70.3)	2949 (67.6)	3535 (74.4)	2929 (70.8)	3259 (79.7)	2381 (79.4)	2531 (85.0)
Low nutritional intake, *n* (%)	11 302 (39.6)	1049 (48.8)	632 (26.8)	2290 (52.1)	1341 (27.2)	2146 (51.9)	1218 (28.2)	1667 (54.5)	959 (30.7)
Smoking, *n* (%)									
Never smoker	16 568 (87.1)	1094 (83.1)	1121 (81.1)	2589 (84.9)	2700 (86.0)	2801 (90.5)	2400 (89.8)	2146 (95.4)	1717 (93.8)
Occasional smoker	447 (2.6)	44 (3.5)	60 (3.7)	110 (3.8)	94 (2.8)	47 (1.5)	52 (1.7)	20 (1.0)	20 (0.9)
Current smoker 0–15 cigarettes/day	1089 (6.4)	117 (8.7)	166 (10.9)	176 (5.9)	231 (7.4)	129 (4.3)	141 (5.0)	62 (2.6)	67 (3.5)
Current smoker 15+ cigarettes/day	690 (3.9)	63 (4.7)	61 (4.3)	176 (5.4)	124 (3.9)	130 (3.7)	84 (3.4)	23 (1.0)	29 (1.7)
Alcohol consumption, mean (s.e.)	3.9 (0.01)	4.1 (0.04)	3.6 (0.04)	4.3 (0.03)	3.7 (0.03)	4.5 (0.04)	3.6 (0.04)	4.4 (0.05)	3.4 (0.05)

We estimated the direct and indirect effects of ACEs on multimorbidity. ACEs, allostatic load, and social engagement significantly predicted multimorbidity (Fig. 2). The number of ACEs was positively associated with the number of chronic conditions, *β* = 0.12 (95% confidence interval 0.11–0.13). Higher social engagement was associated with a decrease in chronic conditions, *β* = −0.10 (−0.12 to −0.08), whereas increased allostatic load was positively associated with the number of chronic conditions, *β* = 0.16 (0.15 to 0.17). In addition to direct relationships, allostatic load and social engagement mediated between ACEs and multimorbidity. Increase in the number of ACEs was associated with lower social engagement, *β* = −0.14 (−0.16 to −0.12) and higher allostatic load, *β* = 0.04 (0.03–0.05), which in turn significantly predicted multimorbidity. The total effect of ACEs (*β* = 0.14; 0.13–0.15) on multimorbidity equals the sum of the direct pathway (*β* = 0.12) and the indirect pathways (*β* = 0.02; Fig. 2).

The relation between ACEs and multimorbidity stratified by sex and age (Table 2) was examined. The results were consistent with those of the overall model; however, the direct effect of ACEs and the indirect effect of ACEs through social engagement and allostatic load on multimorbidity were stronger in females than males. ACEs, allostatic load, and social engagement together accounted for 28% of the variation in multimorbidity among females and 23% among males. Regarding age, the direct and indirect effects of ACEs on multimorbidity were stronger in the younger age group (45–54 years) and weakened with increasing age. Among 75–85-year-old individuals, ACEs had a direct effect on multimorbidity, but the indirect effects via allostatic load and social engagement were not significant. Within this age group, social engagement had a direct effect on multimorbidity, but allostatic load did not. ACEs, allostatic load, and social engagement together accounted for 15% of the variation in multimorbidity in 45–54- and 55–64-year-old age groups, 9% in the 65–74-year-old group, and % in the 75–85-year-old group.

**Table 2.** Pathway estimates from structural equation model for the overall sample and by sex and age groups

Pathway	Males	Females	45–54 years	55–64 years	65–74 years	75–85 years
ACEs→multimorbidity	0.10 (0.08, 0.12)	0.13 (0.11, 0.15)	0.15 (0.13, 0.17)	0.13 (0.11, 0.15)	0.11 (0.08, 0.14)	0.11 (0.08, 0.14)
Indirect	0.01 (0.01, 0.01)	0.03 (0.02, 0.04)	0.03 (0.02, 0.04)	0.03 (0.02, 0.04)	0.02 (0.01, 0.03)	0.006 (n.s.) (−0.002, 0.01)
Total	0.11 (0.09, 0.13)	0.15 (0.13, 0.17)	0.18 (0.16, 0.20)	0.16 (0.14, 0.18)	0.13 (0.10, 0.16)	0.10 (0.07, 0.13)
ACEs→allostatic load	0.04 (0.02, 0.06)	0.05 (0.03, 0.07)	0.06 (0.04, 0.08)	0.03 (0.01, 0.05)	0.03 (0.00, 0.06)	0.004 (n.s.) (−0.03, 0.04)
ACEs→social engagement	−0.11 (−0.13, −0.09)	−0.17 (−0.20, −0.14)	−0.14 (−0.17, −0.11)	−0.17 (−0.20, −0.14)	−0.14 (−0.18, −0.10)	−0.09 (−0.14, −0.04)
Allostatic load→multimorbidity	0.15 (0.13, 0.17)	0.16 (0.14, 0.18)	0.24 (0.21, 0.27)	0.21 (0.19, 0.23)	0.14 (0.11, 0.17)	0.10 (0.06, 0.14)
Social engagement→multimorbidity	−0.07 (−0.10, −0.04)	−0.12 (−0.15, −0.09)	−0.12 (−0.17, −0.07)	−0.13 (−0.17, −0.09)	−0.10 (−0.13, −0.07)	−0.06 (−0.12, 0.001)

All estimates are direct effects unless indicated. Values in parentheses are 95% confidence intervals. n.s., non-significant.

Model adjusted for covariates, viz., income, smoking, nutrition, activity level, and alcohol consumption. Covariance between social engagement and allostatic load was included.

As a final check on the robustness of the model, we analyzed the full-sample data again, using all aforementioned variables, but with the addition of *baseline* multimorbidity as a covariate. The model is significant (Table 3), explaining 73% of the variance (a 47% increase over the model without baseline multimorbidity). All path coefficients are smaller, as compared to the model without multimorbidity, but they are significant. These changes in model parameter estimates are likely due to the stability of multimorbidity, which is increasingly difficult to reverse (Wang et al., 2018); the current data show a bivariate correlation of *r* = 0.82 between baseline and follow-up multimorbidity scores. The addition of the baseline multimorbidity covariate shows that the model's significance is not simply due to illness that preceded the measures of social engagement and allostatic load. Despite the fact that this more inclusive model explains greater variance than does the model without baseline multimorbidity, we use the model without multimorbidity for explanatory purposes below. We do so because we assume that baseline multimorbidity levels are themselves related to ACEs, and to prior social engagement, and allostatic load, consistent with the life course model we propose here. So, although the model that includes baseline multimorbidity accounts for substantially more variance than the one that does not, the additional variance does not reflect the power of the model presented here, but the stability of multimorbidity.

**Table 3.** Pathway estimates from structural equation model for the overall sample, baseline multimorbidity covaried

Pathway	*β* (s.e.)
ACEs→multimorbidity	0.02 (0.004)
Indirect	0.003 (0.0009)
Total	0.02 (0.004)
ACEs→allostatic load	0.02 (0.0006)
ACEs→social engage	−0.13 (0.009)
Allostatic load→multimorbidity	0.05 (0.004)
Social engage→multimorbidity	−0.02 (0.006)
Social engage→allostatic load	–
*R*^2^ for multimorbidity	73%

All estimates represent direct effects unless indicated.

s.e., standard error.

Model adjusted for income, smoking, nutrition, activity level, alcohol consumption, and baseline multimorbidity. Covariance between social engagement and allostatic load was included.

## Discussion

As hypothesized, ACEs were related to multimorbidity and this relation was mediated by social engagement and allostatic load (Fig. 2). To our knowledge, this is the first study to integrate three prominent factors (ACEs, social engagement, allostatic load) into a mechanistic, lifespan model predicting multimorbidity. Both mediators – social engagement and allostatic load – were selected *a priori* because they are sensitive to early adversity and have a central role in physical and mental health across the lifespan (Atkinson et al., 2015). The findings support the theory that each ACE increases stress, undermining social engagement and contributing to allostatic load, thereby increasing the risk of multimorbidity (Anda et al., 2006; Atkinson et al., 2015; Belsky et al., 2017; Berens et al., 2017; Felitti et al., 1998; Nelson et al., 2020; Shonkoff & Garner, 2012).

The model was validated in the overall sample, and in the sex- and age-stratified subsamples (Table 2). Regarding sex, the model pathways from ACEs to both mediators and to multimorbidity, and from mediators to multimorbidity, were consistently stronger in females, compared to males. This may be because females experience more ACEs than do males, as shown here (Table 1, Total ACEs Score 3+) and elsewhere (Nguyen et al., 2019; Tomasdottir et al., 2015), females experience qualitatively different ACEs [females more frequently report sexual abuse, neglect, and household dysfunction (Haahr-Pedersen et al., 2020); males more often report interpersonal violence (McAnee, Shevlin, Murphy, & Houston, 2019)], and females experience greater complexity of ACE co-occurrence patterns (Haahr-Pedersen et al., 2020; McAnee et al., 2019). Therefore, it is likely that females are more vulnerable to adverse outcomes and their precursors than are males (Haahr-Pedersen et al., 2020).

The full model, including pathways between ACEs and multimorbidity, ACEs and both mediators, and both mediators and multimorbidity, was established by age span 45–54. The model remained significant across subsequent age groups (see Table 2 for pathway estimates) but there was a general diminution of all pathways with increasing age. At 75–85, the model remained significant and largely intact, but some pathways were not significant. The direct pathway between ACEs and multimorbidity remained significant, as did the pathways between ACEs and social engagement, social engagement and multimorbidity, and allostatic load and multimorbidity. However, the total *indirect* pathway between ACEs and multimorbidity, through social engagement and allostatic load, was non-significant in this age group. This may be related to the facts that ACEs and mediators are temporally separated and increasingly moderated by intervening events, and multimorbidity inexorably increases with advancing age, regardless of prior influences (Epel, 2020).

However, social engagement remained significantly linked to ACEs and multimorbidity in the 75–85-year age group. The finding confirmed prior observations that the speed of allostatic load and morbidity accumulation was reduced by richer late-life social networks (Dekhtyar et al., 2019), potentially via gene expression (Brown et al., 2020; Rentscher, Carroll, Cole, Repetti, & Robles, 2020) and greater adherence to positive health behaviors (Epel, 2020). Indeed, developmental models, including geroscience models (Epel, 2020), unanimously acknowledge the importance of social engagement across the lifespan (Atkinson, 2019).

It might be argued that the association between ACEs and multimorbidity is simply due to the possibility that ACEs predict common variance shared by chronic disease outcomes. This explanation is unlikely, however. The morbidities comprising the multimorbidity index presented here were largely independent, with a median *φ* coefficient of 0.04, interquartile range = 0.01–0.07. These statistics are consonant with the definition of multimorbidity as the co-occurrence of chronic health conditions, with comorbidities unrelated to an index diagnosis (Nguyen et al., 2019; Wang et al., 2018; Yurkovich et al., 2015). Rather, the relations between ACEs and multimorbidity appeared related to the broad impact of ACEs, social engagement, and allostatic load on diverse and statistically independent morbidities.

The model presented here supports the theory that multimorbidity is a developmental process beginning with early experience and extending across the lifespan. This conceptualization is further supported by the fact that relations amongst ACEs, mediators, and multimorbidity held even after six risk factors, all pertaining to current or relatively late-life conditions (economic challenge, cigarette smoking, alcohol consumption, nutrition, physical activity, and baseline multimorbidity) were covaried in the model. These risks are themselves related to ACEs and chronic disease (Christakis, 2016; Dietz, Douglas, & Brownson, 2016; Ford, Butler, Hughes, Quigg, & Bellis, 2016; Vander Weg, 2011); as such, they comprise particularly stringent covariates.

These data further support recommendations regarding the need for prevention and early intervention in early life (Nelson et al., 2020), when healthy aging apparently begins. It is important to note that social engagement and biological embedding are potently influenced by childhood experience and are increasingly difficult to reverse (Fraley, Gillath, & Deboeck, 2021; Wang et al., 2018). As such, early interventions could forestall the onset of disease across multiple conditions simultaneously (Belsky et al., 2017).

At the same time, interventions at any age are potentially useful (Shonkoff, Boyce, & McEwen, 2009). Social engagement may be particularly important in this regard, as a potentially modifiable factor (Yu, Steptoe, Chen, & Jia, 2021) associated with ACEs and multimorbidity across the full age range assessed here, and may potentially augment interventions aimed at extending the years of healthy function (healthspan; Epel, 2020). Frequently, contemporary interventions involve a ‘whack a mole approach’ (Epel, 2020), comprising *ad hoc*, piecemeal, and in some cases, iatrogenic polypharmacy (Sheikh et al., 2016), with medications prescribed separately by diagnosis (Sathanapally et al., 2020; Sehgal et al., 2013). Supplementation with social interventions (Turner et al., 2016) may exert a more generalized influence, in accord with the World Health Organization's call for simplified multimorbidity management (Sheikh et al., 2016). Moreover, patients with multimorbidity themselves prioritize continued social function, although treating clinicians often do not (Sathanapally et al., 2020).

The model validated here represents a basic platform that can be broadened to encompass additional risk indices and outcomes, mediators, and moderators. Respecting additional mediators, and by way of example, child variables such as self-regulation are central to development and are programmed early (Evans, Fuller-Rowell, & Doan, 2012; Nigg, 2017). Later in life, self-management skills and behaviors, related to adult attachment, quality of relationships with care providers, and chronic disease, may become important mediators (Brenk-Franz et al., 2015, 2017; Ciechanowski et al., 2004). The most effective moderators may involve those which, like the mediators, exert their effects early and broadly. For example, pleiotropic genes like the dopamine receptor *D4* (HGNC:3025) influence diverse neurobiological processes and psychiatric/neurological phenotypes, including reaction to stress (Ptácek, Kuzelová, & Stefano, 2011), central to the allostatic load construct.

The platform might also be expanded via alternative analytic strategies. For example, the current analysis assessed a parallel mediation model, wherein social engagement and allostatic load contributed simultaneously to chronic disease onset. However, an expanded, serial mediation model might better represent the complexity of development (Nelson et al., 2020); or again, the analytic approach used here assessed for mediation and disregarded moderation. But it is possible that social engagement and allostatic load both mediate between ACEs and multimorbidity, and are, at the same time, moderated by ACEs (Hayes, 2017). Indeed, in a post hoc analysis, we found that ACEs moderate the impact of allostatic load (but not social engagement) on multimorbidity. Thus, future research might adopt a counterfactual approach to examine natural, pure, and controlled direct and indirect effects (Hayes, 2017). Considerations presented in the last two paragraphs may augment the platform presented here.

This study has the following limitations. The statistical modeling was correlation-based, precluding unequivocal causality claims. This would require random assignment of participants, experimental manipulation of early life experiences, and very long-term follow-up, impossible with humans. Nevertheless, model results were hypothesized *a priori* based on logic and theory, replicated multiple times across sex and age, and were based on a longitudinal design, all criteria that buttress causal inferences of correlation-based mediation models (Hayes, 2017).

Exposure to ACEs, social engagement, current behaviors, and diagnoses were based on self-report, with potential response bias. Furthermore, ACEs were reported retrospectively. However, the ACEs-outcome link does not appear dependent on the time of reporting, the association between ACEs count and adverse outcomes emerges consistently in both prospective and retrospective studies, and comparisons of data across these studies show no evidence of report bias (Karatekin & Hill, 2019). Importantly, Belsky et al. (2017) found that early life risk factors, assessed prospectively, predicted allostatic load, and they replicated these findings using retrospective self-reports.

Regarding multimorbidity estimates, the use of self-reported clinical diagnoses is common in the epidemiological literature. Within-diagnosis reliability estimates are high (Dal Grande, Fullerton, & Taylor, 2012). Reporting errors typically involve underreporting, and show consistent specificity, albeit variable sensitivity. In any event, the approach provides useful estimates of population prevalence (Chun, Kim, & Min, 2016; Martin, Leff, Calonge, Garrett, & Nelson, 2000). For example, Cho, Chang, Ahn, Shin, and Ryu (2018) found that women underreported smoking but sensitivity and specificity rates of self-reported diagnosis (thyroid cancer) were 98.1% and 99.8%, respectively, when compared to a national registry. Wang and Sungsuk (2019) also showed that smoking was underreported, particularly among women, but the bias did not affect the prediction of cardiovascular disease. Such findings suggest that insofar as bias exists in the current data, it is not sufficient to invalidate findings. This is not to say, by way of proviso, that ACE scores are accurately associated with health difficulties at the individual level; rather, they are related to mean group differences (Baldwin et al., 2021). Nevertheless, clinical trials and interventions for multimorbidity will require quick, inexpensive assessments, such that validation of self-report utility is crucial (Belsky et al., 2017).

In the present study, allostatic load and multimorbidity are assessed 3 years apart. It might be argued that this temporal spacing is insufficient to assess the link between them. However, it is not yet clear what constitutes ideal timing and, in any case, optimal timing may be age-dependent. In earlier adulthood, when allostasis and multimorbidity are less confounded, longer time spans may be more sensitive to cumulating change and gradual disease onset. However, as Pace of Aging (Belsky et al., 2017) accelerates, and allostatic overload and multimorbidity come to coexist as the former progresses into the latter, shorter time spans may better capture the more rapid shifts. This much is consistent with the present data. Allostatic load played a statistically significant mediating role between ACEs and multimorbidity in all age groups except the oldest. The 3-year temporal spacing between measurement of allostatic load and multimorbidity is sensitive to the distinction between these constructs in the younger age groups; it is conceivable that a shorter time span between allostatic load and multimorbidity would have been useful in the assessment of the 75- to 85-year age group.

A related confound involves the fact that social engagement and allostatic load were assessed concurrently, at baseline visit. It is possible that individuals were already experiencing chronic conditions, such that social engagement deficits did not influence multimorbidity, but were influenced by it. We would not deny the likelihood that relations between these processes are transactional (Hostinar, Sullivan, & Gunnar, 2014). However, the mediation model tested here was prespecified (Atkinson et al., 2015), relationality serves a primary function in health and disease across the lifespan, and the measures of engagement used here are highly reliable [The Medical Outcomes Study and Social Support Survey has 1-year retest reliability of *r* = 0.78 in a sample with chronic disease (Sherbourne & Stewart, 1991)]. Moreover, we assessed the impact of multimorbidity at baseline, and the model and all its paths remained significant, suggesting that the history of multimorbidity is not the driving factor in the mediation model proposed here. It is true, however, that a prospective longitudinal study with repeated measures of all constructs and cross-lagged analyses would help resolve ambiguities.

Another limitation of the present study involves the lack of some important covariates and moderators. These include early moderators such as family of origin of medical history, itself linked to multimorbidity (Singh et al., 2019). In addition, features that emerge in the 3 years between the assessment of mediators and multimorbidity [e.g. self-management, relationship between participant and care-providers (Brenk-Franz et al., 2015, 2017)] may mediate or moderate relations between predictors and outcome. Exploration of such variables is recommended in future studies. On the other hand, the present study does control for income, tobacco and alcohol use, diet, and physical activity level, all importantly related to both early adversity and multimorbidity. Furthermore, we conducted an analysis controlling for multimorbidity at baseline assessment. Despite the influence of this extremely potent covariate on multimorbidity at follow-up (baseline and follow-up multimorbidity correlate at *r* = 0.82), the model, and all its paths, remained significant, attesting to its robust nature.

The integrative model presented here is consonant with predictions and consistent across sex and age in a national, population-based longitudinal study. To our knowledge, this is the first empirical demonstration of mediation in the context of ACEs and outcome generally, and ACEs and multimorbidity, in particular. From a clinical perspective, the findings indicate that the knowledge of childhood adversity may serve to identify those at risk for early multimorbidity. The data also suggest that intervention in childhood may reduce the probability of multimorbidity. Furthermore, social engagement facilitated at any age for those at high risk of multimorbidity may reduce or delay negative health conditions. Moreover, the model presented here provides a basic platform that, with expansion, may help identify the life course events and processes that will further explain multimorbidity and provide intervention targets.

## Data Availability

Data are available from the Canadian Longitudinal Study on Aging (www.clsa-elcv.ca) for researchers who meet the criteria for access to de-identified CLSA data.
